# A spiking neural network for active efficient coding

**DOI:** 10.3389/frobt.2024.1435197

**Published:** 2025-01-15

**Authors:** Thomas Barbier, Céline Teulière, Jochen Triesch

**Affiliations:** ^1^ SIGMA Clermont, Centre National de la Recherche Scientifique, Institut Pascal, Université Clermont Auvergne, Clermont-Ferrand, France; ^2^ Life- and Neurosciences, Frankfurt Institute for Advanced Studies, Frankfurt am Main, Germany

**Keywords:** active efficient coding, spiking neural network, event-based cameras, unsupervised learning, reinforcement learning

## Abstract

Biological vision systems simultaneously learn to efficiently encode their visual inputs and to control the movements of their eyes based on the visual input they sample. This autonomous joint learning of visual representations and actions has previously been modeled in the Active Efficient Coding (AEC) framework and implemented using traditional frame-based cameras. However, modern event-based cameras are inspired by the retina and offer advantages in terms of acquisition rate, dynamic range, and power consumption. Here, we propose a first AEC system that is fully implemented as a Spiking Neural Network (SNN) driven by inputs from an event-based camera. This input is efficiently encoded by a two-layer SNN, which in turn feeds into a spiking reinforcement learner that learns motor commands to maximize an intrinsic reward signal. This reward signal is computed directly from the activity levels of the first two layers. We test our approach on two different behaviors: visual tracking of a translating target and stabilizing the orientation of a rotating target. To the best of our knowledge, our work represents the first ever fully spiking AEC model.

## 1 Introduction

For humans and other mammals, learning to see is an active process involving the development of efficient neural representations and accurate eye movements. The Active Efficient Coding (AEC) framework proposes that both aspects are learned jointly to improve the overall coding efficiency of the visual system. This process is thought to occur through a self-calibrating feedback loop where sensory representations continually adapt to the statistics of visual signals sampled by eye movements, while eye movements adapt to enhance the efficient encoding of visual input.

Various AEC models have been proposed to describe the self-calibration of different aspects of vision, such as active stereo vision (Zhao et al., 2012; Lonini et al., 2013; Zhang et al., 2014; Klimmasch et al., 2021), active motion vision (Teulière et al., 2015), accommodation (Eckmann et al., 2020), and torsional eye movements (Zhu et al., 2022), as well as combinations thereof (Lelais et al., 2019). Notably, AEC has also been extended to the auditory domain (Wijesinghe et al., 2021).

However, all these studies have utilized network architectures with continuous activation model neurons. To date, no AEC model has been developed based on Spiking Neural Networks (SNNs). Such a model would be of great interest due to the increased biological plausibility of SNNs and their potential for energy efficiency, especially when sparsity can be exploited in hardware (Christensen et al., 2022; Dampfhoffer et al., 2023). Furthermore, SNNs are naturally suited for processing visual inputs from event-based cameras. These cameras, inspired by the mammalian retina, output a continuous stream of discrete events rather than periodically sampled image frames, offering advantages like low data rates, low latency, and high dynamic range (Gallego et al., 2022).

In this study, we introduce the first fully spiking AEC model. An event-based camera provides input to the model, which comprises a two-layer efficient coding network that learns to represent visual inputs with a minimal number of spikes. A spiking reinforcement learner is trained to control eye movements, aiding in the efficient encoding of visual input. This learner utilizes an intrinsic reward signal generated by the efficient coding network. Consequently, the system learns both neural representations and visual behavior in a fully self-calibrating manner, without requiring external supervision or an extrinsically provided reward signal.

We demonstrate our approach with two visual behaviors: tracking a translating target in two dimensions through pursuit movements of a single camera, and stabilizing the orientation of a rotating target, a problem relevant, for instance, to stabilizing the horizon line during flight. The presented tasks are admittedly quite simple, but they serve as a minimal demonstration of intrinsically motivated reinforcement learning in spiking networks. Better results could be achieved with a carefully designed extrinsic reward signal, but our contribution is intrinsically motivated learning in a fully spiking framework.

## 2 Related work

### 2.1 Learning efficient representations with SNNs

SNNs have been used to take full advantage of the asynchronous nature of event streams. Several studies have modified backpropagation rules in conjunction with supervised learning for SNNs (Ponulak and Kasinski, 2010; Shrestha and Orchard, 2018; Cheng et al., 2020). However, the reliance on extensive amounts of labeled training data makes these approaches impractical for a self-calibrating vision system. Consequently, our focus is on fully unsupervised techniques.

Many researchers have explored the combination of SNNs with the Spike-Timing Dependent Plasticity (STDP) learning rule to learn effective representations. STDP naturally adapts neural representations to match input statistics and has proven effective for digit recognition (Diehl and Cook, 2015; Tavanaei et al., 2018; Iakymchuk et al., 2015; Hopkins et al., 2018). These works demonstrate that a layer of Leaky Integrate and Fire (LIF) neurons, with various homeostatic mechanisms, can produce spike patterns used to classify MNIST digits using Winner Take All (WTA) strategies or additional classifiers. While these visual representations are effective, the applicability and scalability of their networks have not been demonstrated. Moreover, they do not test their networks on real event-based data, instead relying on a conversion of MNIST images to a firing rate representation.

Kheradpisheh et al. (2018) propose a larger convolutional SNN architecture with up to seven layers, inspired by traditional deep learning. They effectively distinguish objects in images using a rate-coding scheme to convert images into spike trains. The complexity of learned representations increases with depth, from Gabor filters in the early layers to parts of objects in the final layers. While these deep spiking architectures are highly performant, they appear not well-suited for closed-loop vision-based control due to their learning speed, energy consumption, and potential processing delays. Our interest lies in more lightweight architectures that can seamlessly integrate with a reinforcement learning agent.

Paredes-Valles et al. (2019) present a multi-layered architecture for estimating optical flow from scenes, learning effective representations based on synaptic delays, and testing on real event-based data. Akolkar et al. (2015) train a SNN using actual event-based data from an event-based camera, learning neuronal receptive fields for classification tasks. Paulun et al. (2018) propose a complex neuronal architecture trained with STDP, representing a model of the primary visual cortex, and test it on the MNIST-DVS dataset.

Chandrapala and Shi (2016) propose a two-layer SNN that learns invariant feature representations similar to simple and complex cells in the brain, using gassom instead of STDP. Their model differentiates digits from the MNIST-DVS dataset with 90% precision, outperforming other event-based processing methods. Debat et al. (2021) designed a three-layer SNN to estimate trajectories, outperforming human capabilities. Guyonneau et al. (2004) show that SNNs can effectively learn efficient visual representations from the spatio-temporal structure of spike patterns, robust to fast visual stimuli, and emphasize the importance of sparsity in neural codes.

Inspired by these principles, we designed our model with a novel combination of homeostatic mechanisms, STDP rules, and architectural features based on prior work (Barbier et al., 2020; 2021). Our approach aims to address the limitations of previous models by developing a more efficient and adaptable vision system.

### 2.2 Spiking reinforcement learning

Learning effective policies using traditional reinforcement learning algorithms is challenging with SNNs due to their asynchronous and spiking nature. Hybrid strategies are often used, where networks are trained using conventional reinforcement learning frameworks and then converted for use with SNNs, as demonstrated by Patel et al. (2019), or where SNNs are combined with traditional deep reinforcement learning for training as in Tang et al. (2020). However, we believe that one can achieve comparable performance using fully spiking-based learning strategies, and demonstrate this approach in here.

The brain’s learning process heavily relies on both extrinsic and intrinsic rewards, similar to how dopamine modulates behavior. The Reward Modulated STDP (R-STDP) learning rule mimics this process by integrating a reward factor with the classic STDP rule. Frémaux et al. (2010); Florian (2007) provide detailed descriptions of R-STDP, which can incorporate rewards directly or through eligibility traces to associate rewards with past actions Gerstner et al. (2018).

While R-STDP has been applied to tasks like MNIST classification Mozafari et al. (2019); Ghaemi et al. (2021), it lacks a traditional reinforcement learning framework involving an agent in a controlled environment. Yuan et al. (2019) address this by combining stochastic and deterministic plasticity learning rules to create neuronal agents, designing the Stochastic-Deterministic Coordinated (SDC) spiking reinforcement learning model, though limited to the design of a logic gate and a simple 19-state random walk application.

Most spiking reinforcement learning models utilize a Temporal Difference (TD) actor-critic framework based on R-STDP. Early works such as Potjans et al. (2009); Jitsev et al. (2012); Nichols et al. (2013) used that framework on simple grid-world and robotic applications.

Weidel et al. (2021) proposed an actor-critic model with a recurrent representation layer and a reward-modulated output layer, validating their model on the mountain car environment. Anwar et al. (2022) created a network for playing a modified pong game, solving 2D visual environments using a spiking reinforcement learning framework. Their network includes a visual cortex for frame transformation, an association cortex for learning visual representations, and a motor cortex for outputting motor commands. The main limitation is the use of a rate-based frame coding approach while we are interested in fully spiking frameworks.

Frémaux et al. (2013); Frémeaux and Gerstner (2016) extended the TD learning framework to a continuous spiking model, using it with a fully spiking actor-critic agent to solve simple maze environments However, their model used fixed-place cells and did not learn efficient representations. Hence, here we extend the framework from Frémaux et al. (2013); Frémeaux and Gerstner (2016), with learnable efficient representation, and apply it to visual environments observed through event-based cameras. Additionally, we aim to learn visual representations that can be directly utilized by the actor and critic populations within the reinforcement learning framework.

### 2.3 Intrinsic reward

One of the biggest limitations of reinforcement learning frameworks is their dependence on externally provided or “extrinsic” rewards. While this can be biologically plausible in certain cases, such as animal experiments where humans deliver food as a reward for successful actions, it is less plausible as a model of how the brain self-calibrates basic sensorimotor loops.

Jaegle et al. (2019) showed that primates heavily rely on visual signals of novelty and curiosity to generate interest and develop specific motor behaviors. Although this is more geared toward higher-level behaviors, such as food search, it suggests that intrinsic motivation plays a significant role in visuomotor learning. It is believed that the human visual system is capable of generating intrinsic rewards based on the efficiency of visual stimuli encoding during eye gaze. Chorley and Seth (2011) attempted to predict dopamine signals using a competitive excitation/inhibition model but did not link this to the learning of concrete visual behaviors.

Gibaldi et al. (2010, 2015) were able to learn vergence control of the eyes using an internal representation of visual input. They utilized disparity-tuned neurons similar to complex cells in the brain to estimate object depth and designed reinforcement learning strategies that used these cells to verge on the target object. Effective gaze strategies were associated with efficient visual encoding and were reinforced over time. This approach was particularly successful for eye vergence, where the left and right visual fields produce very similar stimuli. However, these methods do not operate in the spiking domain and are not applied to event-based visual input. Furthermore, they rely on predefined filters found in the primate visual system and do not learn the efficient coding part.

In this paper, we present a novel approach for generating intrinsic rewards directly from efficient SNN coding layers using plastic synaptic lateral and top-down inhibitory connections. As far as we know, we are the first to propose a fully spiking reinforcement learning framework capable of solving control tasks while intrinsically generating rewards from its internal representation layers.

**FIGURE 1:**
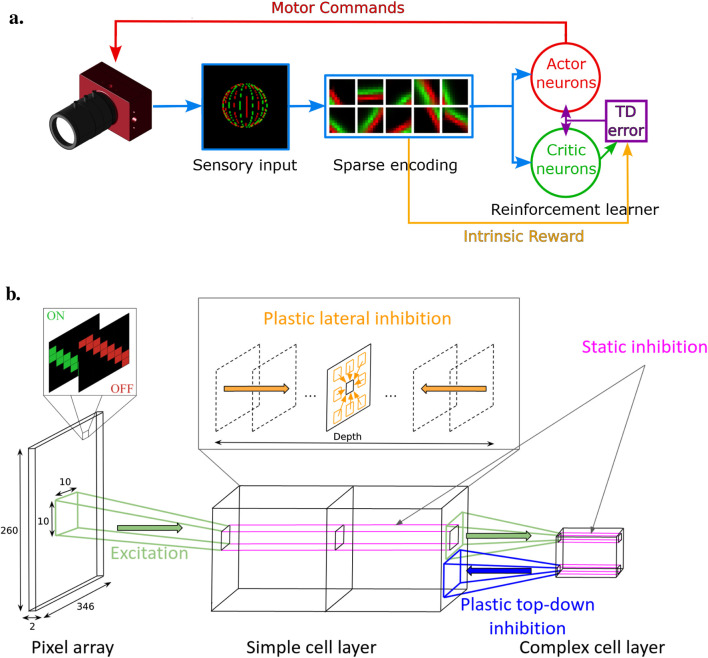
**(A)** AEC spiking model comprising three main blocks: sensory input, efficient coding model, and reinforcement learner. The sensory input from an event-based camera is processed by the efficient coding model to create sparse representations. The reinforcement learner uses these representations to generate motor commands, guided by intrinsic rewards from dynamic inhibition, to optimize visual encoding. **(B)** Sensory input and sparse encoding SNN architecture. The pixel array transmits visual stimuli via ON and OFF channels, which are processed through simple and complex cell layers. Excitation and inhibition mechanisms, including plastic lateral and top-down inhibition, help create sparse, efficient neural representations. Static inhibition stabilizes activity within the complex cell layer. Figure 1B reprinted with permission from N’Dri et al. (2023), Copyright © 2023, IEEE.

Figure 1A illustrates the three stages and main features of our AEC spiking architecture.

## 3 Methods

### 3.1 Efficient coding model

The efficient coding model encodes the visual event stream into a more abstract and compressed representation. We use the efficient coding model described in Barbier et al. (2021). For completeness, we summarize its main elements here. More details regarding homeostatic mechanisms, including refractory periods, threshold and spike rate adaptation, weight normalization mechanisms, as well as weight sharing, can be found in Supplementary Appendix. The model comprises two layers inspired by simple and complex cells in the primary visual cortex of mammals.

#### 3.1.1 Sensory input

Our model is designed to work with event-based data as its input. Event streams are a sparse and asynchronous representation of visual data. Real event-based recordings were captured using a DAVIS346 event-based camera with a resolution of 346×260 pixels. Synthetic event streams were created using traditional videos at high framerate and then converted to event data using the V2E tool Hu et al. (2021). Both ON and OFF events from the event-based camera are fed into the network and handled via separate groups of synapses.

#### 3.1.2 Neuron model

We base all our cells on a LIF neuron model, a simple yet efficient model widely used in SNNs. To complement this model, we include homeostatic mechanisms that regulate spike rates, create inter-cell competition, and increase learning diversity in the network. To limit winner-take-all behaviors where one cell becomes overly active, we added (i) a refractory period and (ii) a spike rate adaptation mechanism. A neuron’s membrane potential then follows:

$$\tilde{V}\left(t+\Delta t\right)=V\left(t\right)e^{\frac{-\Delta t}{\tau_{m}}}-V_{\text{SRA}}\left(t\right)e^{\frac{-\Delta t}{\tau_{\text{SRA}}}}-\eta_{RP}e^{\frac{t_{s}-t-\Delta t}{\tau_{\text{RP}}}},$$ (1)

$$V\left(t+\Delta t\right)=\left\{\begin{array}{cc} \tilde{V}\left(t+\Delta t\right)\; & \text{ : }\tilde{V}\left(t+\Delta t\right)<V_{\theta }\\ 0\; & \text{ : }\tilde{V}\left(t+\Delta t\right)\geq V_{\theta }, \end{array}\right.$$ (2)

where V(t) and $\tau_{m}$ are the membrane potential and the membrane time constant, $V_{\text{SRA}}\left(t\right)$ and $\tau_{\text{SRA}}$ are the spike rate adaptation membrane potential and the membrane time constant, $\eta_{RP}$ and $\tau_{\text{RP}}$ are the refractory trace and refractory time constant, respectively.

#### 3.1.3 Simple cell layer

The first layer of our network aims to approximate the behavior of biological simple cells. These cells learn a more abstract representation of the visual inputs, highlighting features such as orientation, motion, or depth, while also sparsifying the representation.

Our model includes a threshold adaptation mechanism that regulates the membrane potential threshold $V_{\theta }$ according to cell activity S(t) , promoting a more uniform spike rate among all cells.

Synaptic weights are learned using an STDP learning rule. We update the weights $w_{i}$ after each neuron’s spike. The simple cells are connected to the input pixels in their receptive field using two different sets of synaptic weights, one for the ON and one for the OFF events. We track the exact timings of all the presynaptic spikes $t_{i}$ but only the last two postsynaptic spikes $t_{s}$ and $t_{s-1}$ . The weight update is given by:

$$\begin{array}{cc} \Delta w_{i}^{\text{LTP}} & =\eta_{LTP}e^{\frac{t_{i}-t_{s}}{\tau_{\text{LTP}}}}\\ \Delta w_{i}^{\text{LTD}} & =-\eta_{LTD}e^{\frac{t_{s-1}-t_{i}}{\tau_{\text{LTD}}}}, \end{array}$$ (3)

where $t_{s}\geq t_{i}\geq t_{s-1}$ . $\eta_{LTP}$ and $\eta_{LTD}$ control the height of the potentiation and depression windows, whereas $\tau_{\text{LTP}}$ and $\tau_{\text{LTD}}$ control their widths. We use a simple weight normalization mechanism to avoid unbounded growth.

To facilitate rapid and efficient representation learning, our model also includes a weight-sharing mechanism between simple cells. Neurons that look at different locations of the visual field jointly learn the same set of synaptic weights. These neurons belong to the same “neuronal map.” The use of multiple neuronal maps still allows for learning diverse simple cell tunings.

#### 3.1.4 Complex cell layer

Complex cells pool information from multiple simple cells and have larger receptive fields. They learn a higher-level representation that comes from the combination of simple cell receptive fields. We use a different STDP window in the form of a step function with the window centered around the spike time. Both the Long Term Potentiation (LTP) and Long Term Depression (LTD) parts of the window are positive, as follows:

$$\begin{array}{l} \begin{array}{cc} \Delta w_{i}^{\text{LTP,c}} & =\left\{\begin{array}{c} \eta_{LTP}:|t_{i}-t_{s}|\leq \tau_{LTP}\;\\ 0:|t_{i}-t_{s}|>\tau_{LTP}.\; \end{array}\right.\\ \Delta w_{i}^{\text{LTD,c}} & =\left\{\begin{array}{c} \eta_{LTD}:|t_{s-1}-t_{i}|\leq \tau_{LTD}\;\\ 0:|t_{s-1}-t_{i}|>\tau_{LTD}.\; \end{array}\right. \end{array} \end{array}$$ (4)

We do not use weight-sharing here since it would have limited purpose for a pooling layer such as this one. Figure 1B shows the SNN architecture in more detail.

#### 3.1.5 Inhibition mechanisms

Our framework is based on the use of three different inhibition mechanisms, as can be seen Figure 1B that resumes our SNN architecture.

We use a simple static inhibition mechanism to encourage diversity among neurons’ receptive fields. Neurons with the same receptive field location are connected via static inhibitory connections. When a simple (or complex) cell spikes, it inhibits all simple (or complex) cells with the same receptive field location, reducing their membrane potential by a fixed amount:

$$\tilde{V}\left(t+\Delta t\right)=\max\left\{V_{\text{min}},V\left(t\right)e^{\frac{-\Delta t}{\tau_{m}}}-\eta_{I}\right\}$$ (5)

where $\eta_{I}$ sets the strength of this inhibition. This mechanism decorrelates neural responses and facilitates the learning of a diverse set of receptive fields.

Importantly we also use two adaptive inhibition schemes that establish a relationship between the quality of the encoding of the visual input and the amount of spiking activity in the network. This is achieved by learning top-down connections from complex cells to simple cells and lateral connections between simple cells. The rationale is that visual stimuli seen more frequently will trigger more inhibition, thereby reducing network activity for these common stimuli. This activity reduction will be the basis of our intrinsic reward.

In the top-down inhibition scheme, complex cells inhibit all simple cells that excite them via learned connections that instantaneously reduce the membrane potential of the simple cells by an amount corresponding to the learned connection weights. These weights are learned similarly to the excitatory ones, using the STDP rule from Equation 3 applied to inhibitory connections. Simple cells receive inhibitory spikes from the connected complex cells and store them. Once a simple cell spikes, it updates the inhibitory connection weights using a similar STDP window as used for excitatory connections, with the same values for $\tau_{\text{LTP}}$ and $\tau_{\text{LTD}}$ , but different learning rates, $\eta_{\text{ILTP}}$ and $\eta_{\text{ILTD}}$ . A specific normalization factor is applied to inhibitory connections.

**FIGURE 2:**
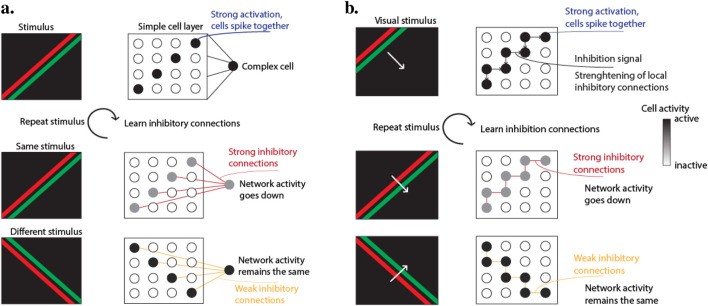
**(A)** Top-down inhibition. Complex cells inhibit simple cells that excite them, strengthening inhibitory connections through repeated visual stimuli, which reduces network activity. **(B)** Lateral inhibition. Simple cells inhibit neighboring simple cells, with inhibitory connections reinforced by repeated visual patterns, thereby decreasing overall network activity for frequent stimuli.

The primary effect of this inhibition mechanism is that cells firing together on a specific visual pattern will inhibit each other, reducing their cumulative spike rate. This effect is illustrated in Figure 2A. For instance, with a moving edge, cells locally close together will experience the pattern simultaneously, driving up their membrane potential and triggering associated complex cells. These complex cells transmit immediate inhibition signals to the simple cells, reinforcing the inhibitory connections with repeated stimuli. After multiple presentations of the same pattern, the inhibitory connections become strong enough to prevent spiking in the set of simple cells, thereby reducing the average activity rate of the network.

The lateral inhibition scheme is similar to the top-down scheme, but acts from simple cells to other simple cells. Designed so that one simple cell inhibits neighboring simple cells, this differs from static inhibition which only works on cells of different neuronal maps sharing the same receptive field location. The same general properties and learning rules as the top-down scheme from Equation 3 apply, with reduced learning rate and normalization factor due to the higher frequency of simple cell spikes.

This lateral inhibition mechanism can be interpreted as a form of predictive learning. When presented with a moving stimulus, like a moving edge, adjacent simple cells activate close in time in a specific pattern. The first set of simple cells activates and sends inhibitory signals to adjacent simple cells. As the edge moves, it activates neighboring simple cells, some of which just received inhibitory signals. These connections are reinforced, as illustrated in Figure 2B. With repeated visual patterns, the inhibitory connections become strong, making simple cells predictors of the pattern. For example, a moving edge triggering the first set of simple cells will strongly inhibit those associated with the next location, reducing total network activity for this pattern. If a different pattern, such as an edge moving in another direction, is presented, network activity remains unchanged since the inhibition does not affect simple cells triggered by this new pattern.

### 3.2 Reinforcement learner

#### 3.2.1 Temporal difference error

Our reinforcement learning agent is based on a TD learning actor-critic framework by Frémaux et al. (2013). The discrete time formulation of the TD learning framework is based on an estimate $V\left(x_{t}\right)$ of the true value function $V^{\pi }\left(x_{t}\right)$ :

$$\delta_{t}=\gamma V\left(x_{t}\right)-V\left(x_{t-1}\right)+R\left(x_{t},a_{t}\right)$$ (6)

with $\delta_{t}$ the temporal difference error at discrete time step t , γ the discount factor, $V\left(x_{t}\right)$ the value function associated to the specific state $x_{t}$ and R the reward associated with the state $x_{t}$ and action $a_{t}$ .

However, in a fully spiking environment, there are no real discrete steps of time t , or if there are (due to the limitation in temporal resolution of event-based cameras), they are too small to be used effectively. Therefore, a continuous TD error is defined as:

$$\delta_{t}=\dot{V}\left(x_{t}\right)-\frac{1}{\tau_{r}}V\left(x_{t}\right)+r\left(x_{t},a_{t}\right)$$ (7)

where $\dot{V}\left(x_{t}\right)$ is the time derivative of the value function, and $\tau_{r}$ is the reward discount time constant that plays a similar role to the conventional reward discount factor.

The TD error gives us an indication of the quality of the value estimate. If the value function perfectly evaluates the real value of a state-action pair, the TD error should be equal to zero. Otherwise, it indicates if the value of a state-action pair is overestimated (negative $\delta_{t}$ ) or underestimated (positive $\delta_{t}$ ).

#### 3.2.2 Critic neurons

The value function estimation is essential to learn a good policy. In SNNs, we cannot evaluate functions as easily as in traditional neural networks. Inputs are arriving continuously, which forces us to look at the evolution of activity in a population of neurons instead.

The first layers of the SNN will serve as a state representation, i.e., their activation patterns represent the state in which the agent is. By connecting those cells to a population of critic neurons, we can learn to associate specific neural activity with a state value function.

Considering one spiking neuron as a value estimator, we can define its value estimate as follows:

$$V\left(s_{t}\right)=\nu \rho \left(t\right)+V_{0}$$ (8)

with $\rho_{t}$ the firing rate of the neuron, $V_{0}$ the baseline for when the neuron has no activity, and ν a scaling factor.

This equation, defines an affine relationship between neuron firing rate and value estimation. Since the firing rate of a neuron is always positive, we can obtain a negative value estimation by carefully selecting $V_{0}$ to be negative. This neuron is called a critic neuron as it performs a similar task as the critic in the discrete formulation.

To make the system more robust, we use a whole population of such critic neurons. Therefore, the Equation 8 can be written as:

$$V\left(s_{t}\right)=\frac{\nu }{N_{\mathrm{critic}}}\overset{N_{\mathrm{critic}}}{\underset{i=1}{\sum }}\rho_{i}\left(t\right)+V_{0}$$ (9)

We usually select a population size of $N_{\mathrm{critic}}=100$ .

The firing rate of neurons evolves continuously over time. An exponentially decaying kernel is used to evaluate the firing rate, defined by:

$$\kappa \left(t\right)=\frac{e^{\frac{-t}{\tau_{k}}}-e^{\frac{-t}{\nu_{k}}}}{\tau_{k}-\nu_{k}}$$ (10)

with $\tau_{k}=100$ ms and $\nu_{k}=5$ ms.

Frémaux et al. (2013) used the derivative of the critic kernel to get an approximation of the value derivative $\dot{V}\left(s_{t}\right)$ . However, we found that the input natural variability of event rates led to instabilities in the derivative. We thus simply use a second-order numerical differentiation on the value, which can be written as:

$$\dot{V}\left(s_{t}\right)=\frac{\eta_{actor}}{N}\overset{t}{\underset{i=N-t}{\sum }}\frac{V\left(s_{i+1}\right)-V\left(s_{i-1}\right)}{2}$$ (11)

with $\eta_{actor}$ a scaling factor and N the number of value points we take into account (since the simulation has a minimal time step of 1 ms).

One limitation of the above formulation is that the value function estimation is proportional to the critic neuron spike rate. Neuronal spike rates are dependent on two main factors: the synaptic weight value of inputs, and the amount of input itself. When submitted to many events, the network’s simple and complex cells will inevitably spike more than when fewer events are present. This, in turn, will drive the critic neurons more, which will impact the value estimation. This is an important difference compared to the work of Frémaux et al. (2013). In their model, they ensure that the amount of activity in the population of state representation spiking neurons stays constant over time. But with event-based visual input, the amount of activity can vary substantially.

To make the value estimation depend only on the values of the weights rather than the event rate, we normalize the value function by the event rate itself. Cell rates in the network are highly correlated with the input rate, which allows us to normalize the event rate without introducing too much variation in the value function estimation.

#### 3.2.3 Actor neurons

In an SNN there are no regular time steps at which actions can be chosen from the output of a readout layer. But similarly to the critic neurons, we can assign spiking neurons to certain actions. In biology, neurons can trigger muscles they are connected to, eliciting fine-tuned movements. Frémaux et al. (2013) used only one actor neuron per action, but to increase stability in the action selection, we use 50 actor neurons for a specific motor action in our framework.

As with the critic neurons, actor neurons are connected to the representation layers so that they can access the agent states’ information. Then, we select actions at regular intervals by looking at the activity of those actor neurons. This process is somewhat of a simplification that is not very biologically plausible. We could envision selecting actions only when a certain amount of activity has been registered in the actor neurons, but that would introduce substantial variability in the control loop. The action selected is the one from the actor population that has the strongest activity. This is a simple WTA mechanism that can be found regularly in biological systems. For simplicity, we do not use a sliding window for computing the firing rate of actor neurons, but instead simply count the number of spikes that occurred since the last chosen action.

#### 3.2.4 Three-factor learning rule

Learning associations between the representation layers and the neurons of the critic and actor is done using the R-STDP learning rule. It is composed of two parts, the traditional STDP rule 3 in addition to a third term, a reward, which is often sparse and will modulate the first rule.

In the traditional STDP rule, we modified the weights every time a neuron spiked. However, this is not possible with a R-STDP rule, as the reward can be sparse and will not always arrive at the same time as the spike of the neuron. For that reason, we only update the weights when the reward signal is transmitted to the neuron.

But we still need to keep track of all the usual changes that the STDP rule would create. To do that, we use synaptic eligibility traces. Each synapse connected to the critic or actor neuron possesses a small eligibility trace that is updated every time the neuron spikes. We apply the usual STDP potentiation and depression to it, in addition to a decay term written:

$$\Delta w_{ei}\left(t+\Delta t\right)=w_{ei}\left(t\right)\frac{e^{-\Delta t}}{\tau_{e}}$$ (12)

with $w_{ei}$ the eligibility trace and $\tau_{e}$ the eligibility decay time constant. As the agent moves in state space, the critic and actor neurons’ eligibility traces will keep track of the neuronal patterns coming from the representations layers. The time constant $\tau_{e}$ determines from how far back in the past information is kept. Then, once the reward is received, we apply the changes to the real weights. For that, we simply multiply the weights with the eligibility traces:

$$\Delta w_{i}\left(t\right)=\eta w_{ei}\left(t\right)\delta_{t}$$ (13)

with η the learning rate and $\delta_{t}$ the TD error that acts as the neuromodulator. With this rule, it is possible for the neurons to learn even with sparse and delayed rewards.

Actions are selected at regular intervals predefined beforehand, which in turn corresponds to the transmission of the reward to the actor and critic neurons. We update both the weights of the critic and actor neurons every time an action is selected, using the current TD error. Importantly, we only update the actor neurons from which the previous action was selected since the TD error estimation arises from that specific action.

If the action contributed to a positive TD error, then we strengthen the actor neurons’ association with the representation layer. In the opposite case, a negative TD error will decrease the weights accordingly.

#### 3.2.5 Exploration and exploitation strategy

Learning both the value and policy at the same time can be difficult. A good policy can only be learned after an effective value approximation has been computed, which requires properly exploring the state space. In this work, we encourage the agent to first explore its environment by selecting random actions regularly, before slowly changing to an exploitation strategy. To allow the network to explore most states at the beginning, the network selects a random action with the probability $\lambda_{\;EXP}$ , called exploration factor. As learning progresses, we decrease this probability, which will drive the agent towards an exploitation phase. In that second phase, the agent has more time to refine its policy.

##### 3.2.5.1 Decay intervals

We handle the change from exploration to exploitation using a simple decay mechanism. Every $\Delta_{decay}$ time interval, we decrease some of the learning parameters, including the exploration factor, action selection interval and critic and actor learning rate η following:

$$\Delta_{\eta }=\left(1-\frac{\Delta_{\eta }\eta_{decay}}{100}\right)$$ (14)

with $\eta_{decay}$ the decay rate.

At the beginning of the learning, the value function has not yet been learned properly. For that reason, it is preferable to reduce the actor learning rate slower than the critic learning rate. We, therefore, use a smaller $\eta_{decay}$ for the actor neurons.

Table 1 details all cell parameters used in our framework. Table 2 presents the reinforcement learning parameters.

**TABLE 1:** Parameters configuration for the network’s cells in the reinforcement learning tasks.

Param	Unit	Simple Cells	Compl- ex cells	Critic Cells	Actor Cells
$V_{\text{thresh}}$	mV	30	3	2	2
$V_{\text{reset}}$	mV	−20	−20	−20	−20
$\eta_{\;LTP}$	mV	0.00077	0.2	0.077	0.077
$\eta_{\;LTD}$	mV	0.00021	0.2	0.021	0.021
$\eta_{\;I}$	mV	15	15		
$\eta_{\;TA}$	mV	1			
$\eta_{\;RP}$	mV	1	1		
$\eta_{\;SRA}$	mV	0.6			
$\eta_{\;ILTP}$	mV	0.0077			
$\eta_{\;ILTD}$	mV	0.0021			
$\tau_{\text{m}}$	ms	18	20		
$\tau_{\;LTP}$	ms	7	20	7	7
$\tau_{\;LTD}$	ms	14	20	14	14
$\tau_{\;RP}$	ms	20	30		
$\tau_{\;SRA}$	ms	100			
$S^{*}$	${sp.s}^{-1}$	0.75			
λ		4	10	4	4
$\lambda_{\text{lateral}}$		100			
$\lambda_{\text{topdown}}$		300			
η	mV			0.2	0.1
$\eta_{\text{decay}}$				5	1.66
$\nu_{\text{k}}$	ms			5	
$\tau_{\text{k}}$	ms			100	
$\tau_{\text{e}}$	ms			250	250

**TABLE 2:** Reinforcement learning framework parameters.

	Action rate	Action rate (min)	$\lambda_{\;EXP}$	$\Delta_{\;decay}$	$V_{0}$	$\tau_{r}$	$\eta_{\;actor}$	ν	N
Unit	ms	ms	%	ms	mV	ms	mV		
Value	250	10	75	2000	−20	1	80	1,000	100

#### 3.2.6 Intrinsic reward from activity

The reward itself is directly generated from the activity of the simple cells. To be precise, we compute a rolling average of the simple cell population activity every 1 ms as follows:

$$S_{t}=\alpha \bar{S_{t}}+\left(1-\alpha \right)S_{t-1}$$ (15)

with $S_{t}$ the activity rolling average, $\bar{S_{t}}$ the averaged sum of all simple cell spikes in that 1 ms window, and α a smoothing factor that we set to 0.75.

Finally the intrinsic reward R is computed as:

$$R=\gamma \frac{\left(\beta -S_{t}\right)}{E_{t}}$$ (16)

with $E_{t}$ the average event rate, which is computed similarly to the simple cell event rate. γ and β are simply scaling factors. We chose γ=5 and β=90 based on experimental results. This reward encourages actions that lead to efficient visual encoding (low activity).

### 3.3 Control on various visual tasks

In this paper, we focus our attention on agents learning to solve a task in a simulated environment. We use CoppeliaSim to create diverse scenarios upon which the agent evolves and acts. It is a robotic simulator coupled with an efficient physics engine. In our case, we used Bullet 2.78 as the physics engine.

By default, CoppeliaSim is not capable of generating event-based camera output. To solve that issue, we capture frames at a very high rate using a small simulation time step of 1 ms. This gives us a frame rate of 1,000 images per second, which we then convert to event streams using an event-based camera emulator called PIX2NVS Bi and Andreopoulos (2018). Even though this is not as precise as real event-based data, the emulator and frame rates are enough to produce accurate models of event streams. Since images are created every 1 ms, we also jitter the events in time to more accurately simulate the output of a real event camera and avoid a cluster of events around frame timestamp generation.

To increase the number of events generated by the moving objects, we added a constant jittering to the camera. This is similar to eye microsaccades and ocular drift, which has been shown to increase visual acuity Intoy and Rucci (2020). It moves left, right, up, and down in saccadic movements, following an Orsnstein-Uhlenbeck stochastic process that can be written as:

$$dx_{t}=\theta \left(\mu -x_{t}\right)dt+\sigma dW_{t}$$ (17)

where θ>0 and σ>0 are parameters that control, respectively, the attraction and drift components. μ is the central point to which the system goes back. $W_{t}$ denotes a Wiener process, a stochastic value generator. Similar jittering techniques have been implemented in Ahissar and Arieli (2012); Testa et al. (2023). The idea behind this jitter is that the camera will continuously drift from the central point μ due to the Wiener process, while being attracted back to it over time.

We created two different environments to test our framework, each having specific properties and challenges.

#### 3.3.1 Tracking task

**FIGURE 3:**
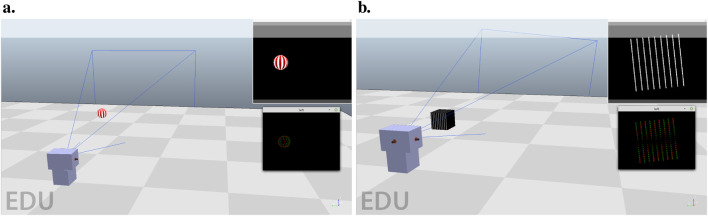
**(A)** Tracking environment, composed of one motorized agent (gray boxes) and one ball (with white and red stripes). The goal is to bring the ball to the center of the visual field, as seen in the top right of the image. Simulation images are sampled at high frame rates and then transformed into event streams, as seen under the visual field representation. The agent can either turn right or left in the horizontal plane. **(B)** Stabilization environment, composed of one motorized agent (gray boxes) and a grating stimulus of white bars on a black background. The goal is to align the bars to a horizontal orientation by rotating the camera around its optical axis.

The tracking task consists of one motorized camera with one axis of rotation. Figure 3A presents a screenshot of the environment. A textured ball is moving in a circle around the camera, whose radius is 1.3 m from the optical center of the camera. It is either moving clockwise or counter-clockwise. The goal of the task is simple, to be able to track the ball by keeping it in the center of the visual field. The simple cell layer is composed of a thin strip of 30 neurons in width and 6 neurons in height that covers the entire ball. We specify the parameters of the architecture in Table 3.

**TABLE 3:** Connectivity parameters. The values indicate dimensions of the following form: (x×y×z) cells.

	Simple cells		Complex cells		Critic cells	Actor cells
Retina size	# cells	Receptive field	# cells	Receptive field	# cells	# cells
Network architecture for the tracking task						
(300×60×2)	(30×6×64)	(10×10×2)	(10×2×16)	(3×3×64)	(100)	(100)
Network architecture for the stabilization task						
(160×160×2)	(16×16×144)	(10×10×2)	(4×4×16)	(4×4×144)	(100)	(100)

#### 3.3.2 Stabilization task

The stabilization task also consists of one motorized camera. The environment is shown in Figure 3B. This time, the rotation is performed around the camera’s optical axis. This task tries to simulate in a simplified way the process of a flying insect that would have to keep its flight horizontal using visual cues. We generate the visual cues from a set of straight bars. A leveled flight corresponds to the bars being horizontal.

This task is harder than the previous one since the network must be able to process the bar orientation to effectively distinguish between states. We will demonstrate in Section 4.1 that the network cells can efficiently differentiate between oriented stimuli, which makes them effective state representation for such a task. The actions are rotating clockwise or counter-clockwise to bring back the bars to the desired horizontal state.

We changed the neurons’ spatial arrangement for that task to be able to fit the entire visual stimulus in the visual field of the network. The stimulus is located in a square in the center of the visual field. We summarize the parameters of the network architecture in Table 3.

## 4 Results

In this section, we evaluate separately the different stages that compose our AEC model, namely the efficient coding model, the intrinsic reward calculation, and the reinforcement learner.

### 4.1 Efficient coding model

**FIGURE 4:**
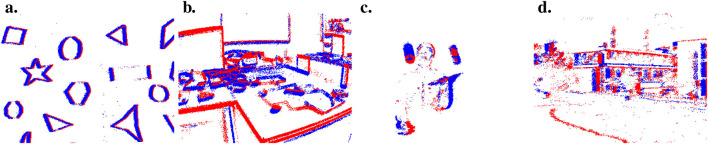
Screen capture of four event recordings, **(A)**shapes on a sheet of paper, **(B)**view of an office, **(C)**someone juggling, and **(D)**view from a robotic platform in an urban environment.

The efficient coding model is the foundation on which the whole AEC model rests. As discussed in Section 3.1.1, events are mostly triggered by the moving edges of objects. An edge can be characterized by different properties, such as its orientation, speed, or disparity in the case of stereovision. A good coding representation must be able to capture all this information while removing unnecessary and redundant parts of the information itself. We showed in Barbier et al. (2020, 2021); N’Dri et al. (2023) that our network is able to learn simple and complex cell-like receptive fields based on synthetic and natural event-based recordings, such as the ones presented Figure 4.

Here, we study the simple and complex cell responses to various types of event inputs. We selected four event recordings in different environments: shapes drawn on a sheet of paper moving in front of the sensor, a recording of an office, someone juggling with balls, and a mobile robotic platform in a small recreated urban road environment. Each recording is a few seconds to a few minutes long.

We created a network of similar architecture than the one presented in Table 3 for the stabilization task. We did not add actor and critic cells since we are not interested in motor control for this specific experiment. We first trained the network on the shapes recording, then we fed the four event recordings to the network and recorded the cells’ activity. The shape data contain edges of multiple orientations, speeds, and widths, so that the network can efficiently learn a wide representation of oriented filters.

Event streams are already sparse by nature, but still contain up to millions of events per second. The simple cell layer, due to the integrating nature of simple cells, reduces that number by a huge factor. We recorded the number of spikes for the simple and complex cell layers, as well as the difference between the normalized event activity and the normalized simple cell activity. We present those results in Table 4.

**TABLE 4:** Sparsity analysis on four different event recordings. We show the result for a network with learned representation. We present the activity reduction, the number of events divided by the number of spikes in the cell layer. We also present the correlation coefficient between the events and simple cell activity.

Scene	Urban	Office	Shapes	Juggling	Mean
# of events	11, 8M	3, 6M	6, 5M	27, 9M	
Simple cell activity reduction	173	112	67	112	116
Complex cell activity reduction	983	610	623	410	657
Simple cell correlation coefficient	0.84	0.90	0.98	0.92	0.91

We measure the activity reduction induced by the efficient coding layers as the number of input events divided by the total number of spikes of the layer. Here, on average, the trained efficient coding layer is able to reduce the input activity by a factor of 116 for the simple cell layer and 657 for the complex cell layer.

To verify that the encoding by the simple cell layer reflects the amount of input events we also computed the correlation coefficient between the number of events and the simple cell activity. These correlation coefficients are given in Table 4. We can observe that the correlation between the input and simple cell layer is very high, especially for the shapes recording that was used for learning the representations.

We showed in previous work N’Dri et al. (2023) that our network is capable of retaining essential information from the visual input stimuli. There we used a classical neural network approach to reconstruct specific parameters from the activity of the simple cells layer. Information such as position, width, length, orientation and movement direction from a set of generated moving bars was correctly predicted, ranging from 80 to almost 100% accuracy depending on the type of information reconstructed. We refer the reader to the mentioned article for more detail on that experiment.

### 4.2 Intrinsic reward calculation

To generate an intrinsic reward signal, we rely on the ability to reduce network activity via a form of learned inhibition. This inhibition reduces network activity for frequently observed stimuli. To demonstrate that, we learn with specific well-controlled input statistics and the results show that the network activity is inversely related to the frequency of observed input patterns. Here, we are using the tracking simulation environment to generate stimuli. We show the network a repetition of a short recording that involves the ball moving back and forth around a specific part of the visual field. Since the network should show reduced activity for central visual stimuli, we need to expose the network to a majority of visual patterns happening in the center of the visual field. For that purpose, the position of the ball in the visual field is selected according to a normal distribution whose mean is located at the visual center. That way, we make sure the network is mostly exposed to visual inputs that will excite the center cells, which in turn will drive up the inhibitory connections between those cells. With enough repetitions, the network starts to learn to strongly inhibit inputs at the center of the visual field, while not so much on the borders of the visual field. In the first experiment, we fix the excitatory weights and only learn the top-down and lateral inhibitory connections.

**FIGURE 5:**
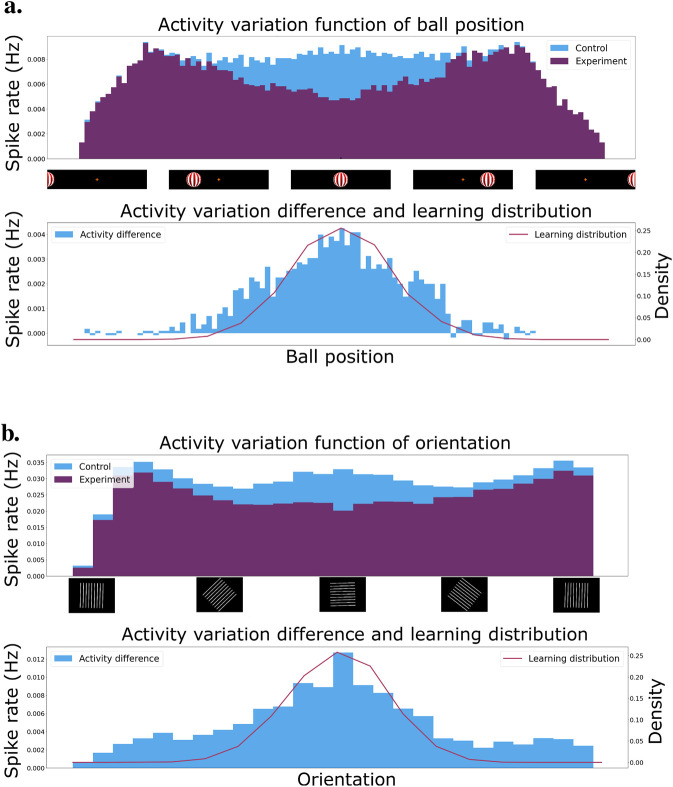
**(A)** Activity of the control and experiment network when presented to a ball moving from the left to the right of the visual field. In purple, the experiment network after learning the inhibition on a normal distribution of ball positions centered on the middle of the visual field. In blue, the control network is the experiment network without inhibitiory weights. The bottom graph presents the normal distribution used for learning in red superimposed on the activity difference between the control and the experiment network. **(B)** Activity of the network when presented to oriented gratings from 0 to 360 ° . In purple, the experiment network after learning the inhibition on a Gaussian distribution of oriented gratings centered around 0 ° . In blue, the control network is the experiment network with shuffled inhibition weights. The bottom graph presents the Gaussian distribution used for learning in red superimposed on the activity difference between the control and the experiment.

Figure 5A presents the result of a single recording of the ball moving from the left to the right of the entire visual field at the end of learning. We recorded the total cell activity and present it as a histogram of activity over time. In blue, we have the control network, which is the network with shuffled inhibitory weights. In purple, the experiment network with inhibitory weights learned on the distribution mentioned above. The distribution can be seen in red in the bottom graph. The latter also presents the activity difference between the control and experiment network.

The activity of the experiment network drops significantly when the ball approaches the center of the visual field. More importantly, the drop is gradual. This means we can use the activity of the network as an effective intrinsic reward signal. Low activity implies a high reward, whereas high activity implies a low reward. The activity of the networks drops when the ball exits the visual field on the right or left since there are much fewer cells to excite in those regions. This does not pose a problem since we normalize the intrinsic reward by the number of events.

For the second experiment, we consider the stabilization task environment. Again, the excitatory connections are fixed and we only learn the lateral and top-down inhibitory connections. We present oriented gratings to the network whose orientations are drawn from a normal distribution centered around the horizontal orientation. The first graph in Figure 5B shows the network activity as a function of grating orientation. The distribution of orientations is shown in red in the bottom graph, along with the activity difference between the experiment and control conditions.

We observe that the activity of the network decreases significantly close to the center of the distribution, i.e., for horizontal stimuli. This is represented as a bigger activity difference between the control and experiment network. Furthermore, the decrease in activity is gradual as we change the orientation. This means we can extract a smooth reward directly from the network activity and use it for reinforcement learning.

### 4.3 Reinforcement learner

#### 4.3.1 Learning the tracking task

We consider a simple scenario with only two possible actions, moving the camera to the right or to the left. We used 100 critic neurons, as well as 100 actor neurons, 50 for each action. These cells receive inputs from the simple and complex cell layers.

For this task, we first learned the weights of the efficient coding layer and fixed them to focus on the reinforcement learning framework. We learned a diverse basis similar to the one presented in Section 4.1. We start in full exploration mode, where every action is selected randomly. During that phase, only the weights of the critic neurons are updated. Every 2 s of simulation time, we decrease both the exploration rate, critic and actor learning rate as well as the time between two action selections. As we continue to decrease the exploration rate, the actions selected are less and less random, and the network fine-tunes its policy.

**FIGURE 6:**
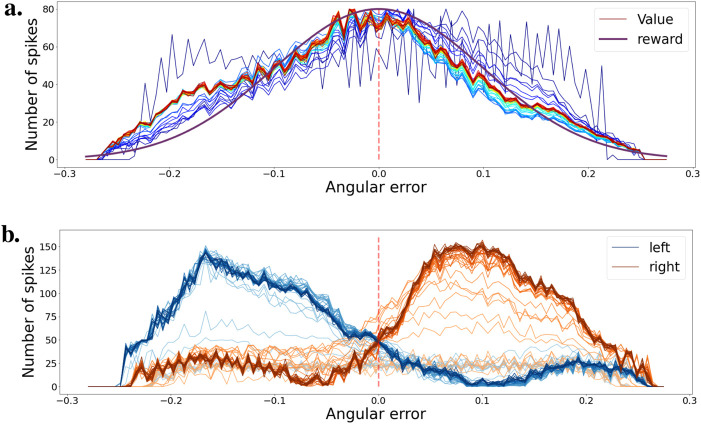
**(A)** Evolution of the value function during training for various ball positions. Color progression from blue (early training) to red (late training) indicates training stages. The purple curve represents the learning distribution used for learning the intrinsic reward. **(B)** Evolution of the agent’s policy during training, shown as actor cell spike rates. Color intensity varies from light (early training) to heavy tones (late training). Actions for left and right movements are depicted in blue and orange, respectively.

We recorded the weights of the network every 2 s in the simulation. Then, we observe the evolution of the network’s performance in a simple validation task. The tracking environment consists of moving the ball back and forth once from the left to the right part of the visual field. For each location, we accumulate spikes from both critic and actor neurons to calculate the estimated value and policy. We submit the network to this validation for every recorded network checkpoint during training. We observe the change in value and policy as the network learns the task in Figure 6.

Figure 6A shows the evolution of the value function over the course of the whole training. Evolution over time is represented by a shift in color from blue to red. In the beginning, the value function is flat and noisy, but very quickly, over a few simulation seconds, it learns to associate a high value to states with a small angular error. As the learning rate decreases, the value stabilizes. The value function is somewhat representative of the learning distribution used in Figure 5A to generate the intrinsic reward, represented here in purple.

Figure 6B shows the policy evolution during training. The blue and orange curves correspond respectively to the left and right motor action. If the ball is in the left part of the visual field, the agent must rotate the camera to the left to bring the ball back to the center. The opposite is true if the ball is in the right part of the visual field. The blue and orange curves show the actor’s spike rates for the different ball positions in the visual field. As training progresses, the network learns to select the correct action depending on the position of the ball. The evolution of the policy over time during training is represented by the color intensity in the curves, from light to deep tones.

In this environment, the network was able to learn an effective policy in a very short time. After only a minute of training, the network easily differentiates the different states of the ball and can select the correct action accordingly.

**FIGURE 7:**
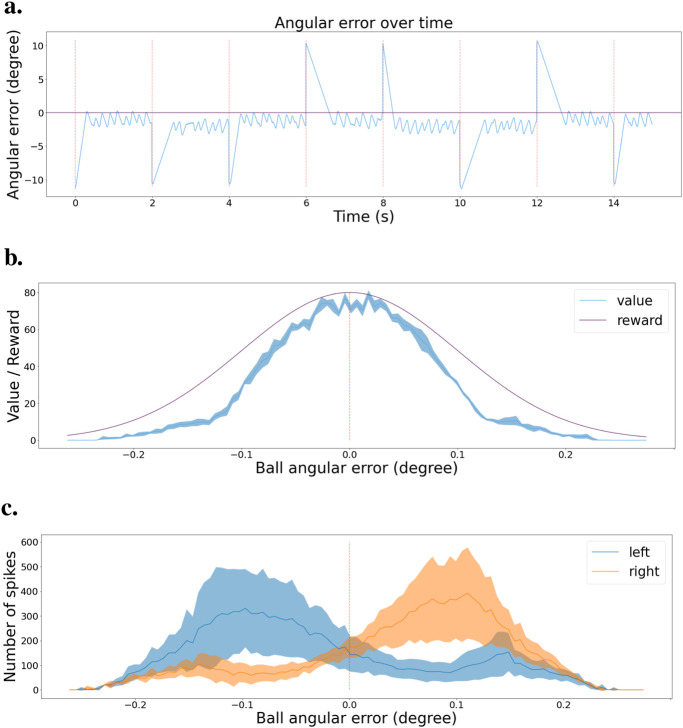
Validation scenario for the tracking task **(A)** Angular error is plotted as a function of time during the validation scenario. Red dashed lines indicate the times when the ball is reset to a random location. Zero angular error corresponds to the ball being in the center of the visual field. **(B)** Value function at the end of learning, with mean and error bands of 1 standard deviation from five experiments with different starting seeds. The purple curve represents the learning distribution used for learning the intrinsic reward, shown for visual comparison. **(C)** Agent’s policy at the end of learning, depicted as actor cell spike rates with mean and error bands of 1 standard deviation from five experiments with different starting seeds. Left and right actions are shown in blue and orange, respectively.

To estimate network performance, we tested the final policy of the network in the simulated environment. The network selects actions every 10 ms, and every 2 s, we reset the ball to either the left or right border of the visual field. We keep track of the angular error between the center of the visual field and the center of the ball. Figure 7A shows the resulting error during the test. We observe that the network successfully brings back the ball to the center of the visual field every time the ball is reset, represented by a red dashed line. Then, the network keeps the ball in the center. We can note that the tracking is not perfectly stable, as there is some jittering when trying to keep the ball in the center, but in general, the policy is effective.

We submitted the network to a validation scenario similar to that when using two actions. The ball is going back and forth from left to right, then right to left. We kept track of the spike train of both the critic and actor neurons and created a histogram of activity based on the angular error from the ball to the center of the visual field. Figure 7B presents the critic histogram in blue, while the purple curve represents the learning distribution used to generate the intrinsic reward. We can see that the activity of the critic neurons tends to match the learning distribution. Figure 7C shows the activity of the two subgroups of actor neurons, 1 for each action. When the ball is in the left part of the visual field, the actor neurons associated with the turning left action spike the most. On the contrary, the actor neurons linked to the turning right action spike more when the ball is on the right part of the visual field. The learned policy is sensible and allows the network to efficiently track the ball in any situation.

#### 4.3.2 Learning the stabilization task

**FIGURE 8:**
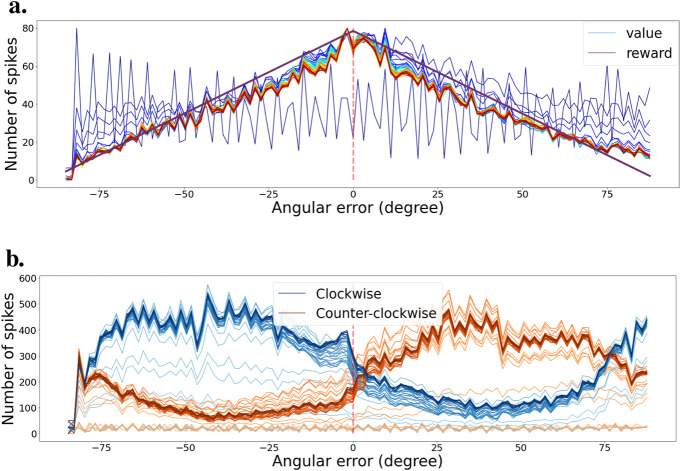
**(A)** Evolution of the value function during training for various bar orientations. Color progression from blue (early training) to red (late training) indicates training stages. The purple curve represents the learning distribution used for learning the intrinsic reward. **(B)** Evolution of the agent’s policy during training, shown as actor cell spike rates. Color intensity varies from light (early training) to heavy tones (late training). Actions for clockwise and counter-clockwise movements are depicted in blue and orange, respectively.

Just like for the tracking task, we also learned the efficient coding layer independently first. We also use a similar exploration and exploitation learning strategy. We saved the weights at regular intervals during training and present the results in Figure 8. Figure 8A demonstrates that we learn an efficient value function during training. Very quickly, the value starts to reflect the learning distribution that was used for generating the intrinsic reward as seen in Figure 5B. Similarly, Figure 8B shows that the actor neurons learn to separate the states as training progresses.

**FIGURE 9:**
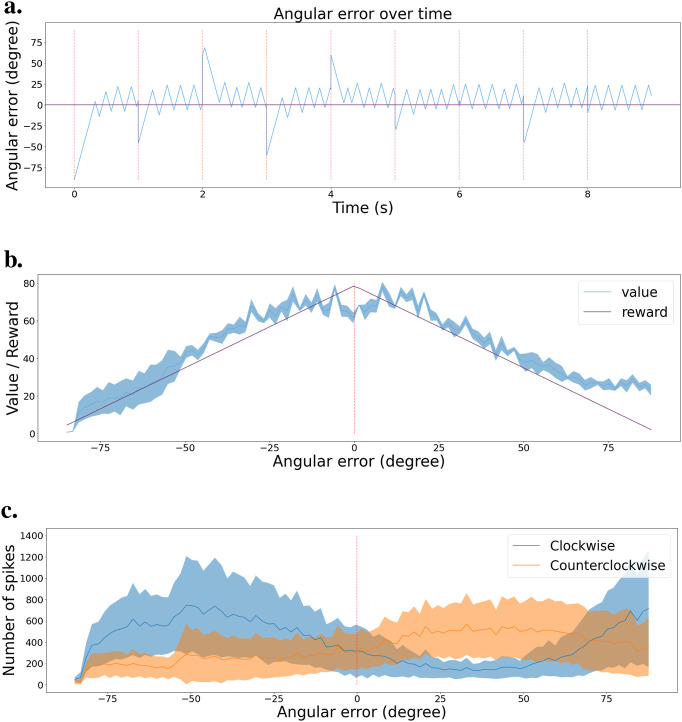
Validation scenario for the stabilization task. **(A)** Angular error is plotted as a function of time during the validation scenario. Red dashed lines indicate the times when the camera’s rotation is reset to a random angle. Zero angular error corresponds to maintaining the grating horizontal. **(B)** Value function at the end of learning, with mean and error bands of 1 standard deviation from five experiments with different starting seeds. The purple curve represents the learning distribution used for learning the intrinsic reward, shown for visual comparison. **(C)** Agent’s policy at the end of learning, depicted as actor cell spike rates with mean and error bands of 1 standard deviation from five experiments with different starting seeds. Clockwise and counter-clockwise actions are shown in blue and orange, respectively.

We also recorded the angular error between the actual and optimal bar orientation (horizontal) during an exploitation test scenario on the fully trained network. Actions are selected every 10 ms. Figure 9A presents those results. The bar orientation is reset every second at a random orientation. We can observe that the network can bring the bars to the right orientation no matter the initial angle. Once in the right orientation, the network can keep the bars horizontal most of the time, even though it presents a small oscillating behavior.

Then, similarly to the tracking task, we designed another simple validation scenario to observe the value and policy of the network when changing the bar orientations. We start with bars at −90 ° orientation (vertical in our case), then rotate them to +90 ° and back again to −90 ° . That way, the network observes all possible orientations in both rotation directions. During that test, we deactivated the actions and recorded the spike trains of the critic and actor neurons.

Figure 9B shows the activity histogram of the critic neurons. We averaged the clockwise and counterclockwise rotations together for more accuracy. The value function effectively associates a higher value to states with a smaller angular error. Figure 9C presents a similar representation but for the actor neurons. We can see the switch in which actor neurons spike the most around the horizontal orientation (0 ° ). We note that the decision boundary is slightly shifted to the right, which can also be observed in Figure 9A. This shows that our spiking reinforcement learning framework works well overall, but might lack resilience for learning a precise policy on more complicated visual tasks.

## 5 Discussion

We have presented the first spiking neural network implementation of the AEC approach. From visual sensing with an event-based camera all the way to motor output, everything works in the spiking domain. Our network autonomously learns simple visual behaviors. The efficient coding component learns efficient visual representations in an unsupervised manner, while the reinforcement learning component uses an intrinsically generated reward based on the spiking activity of the efficient coding component. Thus, the method requires neither supervision nor an externally provided (“extrinsic”) reward signal.

We demonstrated the feasibility of the approach with two admittedly very simple tasks: tracking a moving object through pursuit movements or stabilizing the orientation of a rotating (horizon) line. As such, our work should be viewed as a proof of concept rather than a highly performant solution to these particular tasks. It should be noted, however, that AEC is quite general and has been applied to various active perception skills in the past including active stereo vision Zhao et al. (2012); Lonini et al. (2013); Klimmasch et al. (2021), active motion vision Zhang et al. (2014); Teulière et al. (2015), accommodation control Eckmann et al. (2020), torsional eye movements Zhu et al. (2022) and combinations thereof Lelais et al. (2019) as well as echolocation in bats Wijesinghe et al. (2021). This suggests that our spiking approach to AEC could be extended to any of these behaviors and possibly more.

A limitation of the approach presented here is that in order to establish the intrinsic reward signal, we enforced a particular distribution of sensory inputs (ball positions or grating orientations) during learning of the lateral and top-down inhibitory connections. Therefore, the current approach does not learn fully autonomously. This is in contrast to previous (non-spiking) AEC models which did not require such interventions.

Many parts of our model are inspired by findings on the mammalian brain, such as the presence of simple and complex cells in visual cortex or the utilization of temporal difference learning. However, the model is not fully biologically plausible. Some obvious limitations in this respect are the use of instantaneous synaptic transmission in excitatory or inhibitory connections or the violation of Dale’s law, i.e. single units in our model can both excite and inhibit other units, which does not seem to be the case in biological neural networks. Also, the reinforcement learner’s action choices are based on an external clock and sampled at regular intervals. We believe that some of those limitations with respect to biological plausibility could be overcome relatively easily, but this was not our focus.

Since our model is formulated entirely as a spiking neural network, it would be interesting to implement it on neuromorphic hardware Javanshir et al. (2022); Rathi et al. (2023). This could speed up computation and allow learning visual behaviors in real-time, opening the door to real-world applications of the approach.

## Data Availability

The datasets presented in this study can be found in online repositories. The names of the repository/repositories and accession number(s) can be found below: https://github.com/comsee-research/Neuvisys.
