# Supplementary material for: A spiking neural network for active efficient coding
Source: Front Robot AI. 2025 Jan 15;11:1435197. doi: 10.3389/frobt.2024.1435197 (PMC11775837; doi:10.3389/frobt.2024.1435197)
Supplement: Supplementary file 1 [file Supplementaryfile1.pdf]

APPENDIX

Additional details on the method

Homeostatic mechanisms

The LIF neuron presented before is one of the simplest models. But biological neurons exhibit much more complex mechanisms to adapt their behavior to varying situations. We replicate some of the most beneficial of those mechanisms in order to improve the stability, adaptability and robustness of our spiking neural network.

Refractory period

When a neuron spikes, it enters in a period of low excitability called a refractory period. This strongly limits the spike frequency of neurons in the case of frequent and/or large inputs. We model a refractory mechanism through a trace η_{RP} generated after each spike and then decaying exponentially back to zero at a rate defined by τ_{RP}. The membrane potential update becomes:

$$\tilde{V}(t+\Delta t) = V(t)\, e^{\frac{-\Delta t}{\tau_m}} - \eta_{\mathrm{RP}}\, e^{-\frac{t+\Delta t - t_s}{\tau_{\mathrm{RP}}}}\,, \tag{1}$$

where t_s is the time of the neuron's last spike.

Threshold adaptation

Event-based cameras present an inherent variability in the output frequency depending on factors such as lighting conditions, the amount of textures or the relative speed of objects. This means that neurons will be subjected to variable data rate during their operation, which in turn can lead to high variability in their spike rates. Although this is somewhat unavoidable, it is preferable to keep the spike rate of neurons in a reasonable range across different conditions. Biology handles that problem by means of a wide range of homeostasis mechanisms, be it at the heart of the neuron, the soma, or at the sites of its inputs, the synapses.

We implemented two such mechanisms. The first one is oriented towards long term regulation. It constantly balances the value of the threshold V_θ depending on the spiking activity $S(t)$ of a neuron in order to reach a target spike rate S^*. It can be written as:

$$\Delta V_\theta = \eta_{\mathrm{TA}}\, (S(t) - S^*)\,, \tag{2}$$

with η_{TA} controlling the speed at which the threshold adapts. $S(t)$ is computed by counting the number of spikes which occurred in the previous 10 seconds. The threshold update happens every second, and is therefore a somewhat slow process intended to handle global illumination and speed conditions. We define a minimum threshold $V_{\theta\,\mathrm{min}}$ to avoid capturing camera noise in areas with very little inputs.

Spike rate adaptation

For local variations, we use a faster process called spike rate adaptation. Just like the threshold adaptation, it relies on the neuron's activity. When a neuron spikes, a trace $V_{\mathrm{SRA}}(t)$ is increased by a value η_{SRA}. This trace is subtracted for each pre-synaptic input and decays exponentially back to 0 according to the

parameter τ_{SRA}. The membrane potential internal update becomes:

$$\tilde{V}(t+\Delta t) = V(t)\, e^{\frac{-\Delta t}{\tau_m}} - V_{\mathrm{SRA}}(t)\, e^{\frac{-\Delta t}{\tau_{\mathrm{SRA}}}} \tag{3}$$

This regulation mechanism acts immediately after a spike has happened. By choosing a relatively small time constant τ_{SRA} (in the order of a few hundreds of milliseconds), the effects of the spike rate adaptation mechanism are only visible on short periods of time, which complements the slower threshold update.

Weight normalization

The STDP rule can lead to a great disparity between weights and instability due to unbounded growth. We introduce a weight normalization mechanism in order to avoid this and to control the strengths of different connection types in the network. This aims to model a limited supply of synaptic building blocks in a simple fashion. Let $\tilde{W}$ be a non normalized weight vector of a neuron, obtained after a weight update via STDP. The weights W are then updated according to:

$$W \leftarrow \lambda \frac{\tilde{W}}{\|\tilde{W}\|}\,, \tag{4}$$

where λ is a scaling factor. The aforementioned equation is applied to all weights (excitatory and inhibitory) of our network for all neurons. The value of λ varies according to the type of connection.

Weight sharing

In order to extract an identical set of features with groups of simple cells representing different retinal locations, simple cells share their excitatory weights as in convolutional neural networks. Note that the rest of the weights in the network are not shared but individual. In particular, we did not apply any weight sharing mechanism for complex cells, so that the features extracted become tuned to the specific inputs at that spatial location.

We provide our code for replicating our study in the following github: https://github.com/comsee-research/Neuvisys.
